# A Real Headache: Intracranial Extension and Epidural Abscess As Complication of Chronic Mucocele

**DOI:** 10.7759/cureus.49875

**Published:** 2023-12-03

**Authors:** Divya Naik, Kristopher Aten, Dylan Lopez, Jaimin Patel

**Affiliations:** 1 Internal Medicine, Methodist Health System, Dallas, USA

**Keywords:** chronic sinusitis, rhinorrhea, intracranial extension, cocaine use, epidural abscess, mucocele, case report

## Abstract

Mucoceles are benign lesions of salivary glands typically originating from the paranasal sinuses. Intracranial extension and superinfection of these lesions are rare but serious complications of chronic mucoceles. Here, we discuss a patient with a known mucocele, initially lost to follow-up, who presented three years later with headache, purulent rhinorrhea, and intracranial extension of his mucocele with development of an epidural abscess. This case highlights the potential complications of chronic, large mucoceles and emphasizes the importance of thorough evaluation in patients with facial abscesses in the setting of known sinus pathology. Any mucocele with signs of superinfection such as purulent rhinorrhea, abscess near the sinuses, or refractory symptoms should warrant cranial imaging. Mucoceles with evidence of intracranial extension require neurosurgical and/or otolaryngologic evaluation for evacuation and debridement to avoid neurologic injury or devastating infection.

## Introduction

Mucoceles most frequently involve the frontal and ethmoid sinus, and sometimes the maxillary sinus [1]. They commonly develop in patients with chronic sinusitis, those with facial bone abnormalities, or those with previous trauma or injury [2,3]. Endoscopic sinus surgery has become the new standard for simple mucocele removal [4,5]. Patients who are unable to have mucoceles removed are at risk of developing complications, such as proptosis, diplopia, numbness, ataxia, painful headaches, purulent rhinorrhea, sinusitis, and facial swelling [6]. Rare, but serious, complications include intracranial extension, frontal lobe syndrome, and the development of abscesses or mucopyocele [6]. This case highlights a patient with a mucocele who was lost to follow-up and subsequently developed intracranial extension as well as an epidural abscess.

## Case presentation

A 67-year-old male with a past medical history of chronic sinusitis and a known frontal sinus mucocele who was lost to follow-up presented to the emergency department with complaints of a forehead mass, facial pain, and nasal drainage. He endorsed symptoms for two weeks and reported he did not seek medical care at the onset of swelling due to lack of pain at the time. On admission, he complained of sharp forehead pain and occasional clear nasal drainage with some streaks of blood. The mucocele was initially diagnosed three years prior with a CT scan when the patient was admitted to the emergency department with an exacerbation of his chronic sinusitis symptoms with ataxia per review of prior records. The plan then was to pursue outpatient surgical evacuation; unfortunately, the patient was lost to follow-up. The patient’s past medical history also included a remote history of stroke, thirty pack-year smoking history, cocaine use, and marijuana use. He did not have any history of hypertension, diabetes, or major cardiac events. Prior surgical history included ORIF of the ankle joint following foot trauma a few years prior.

**Figure 1:**
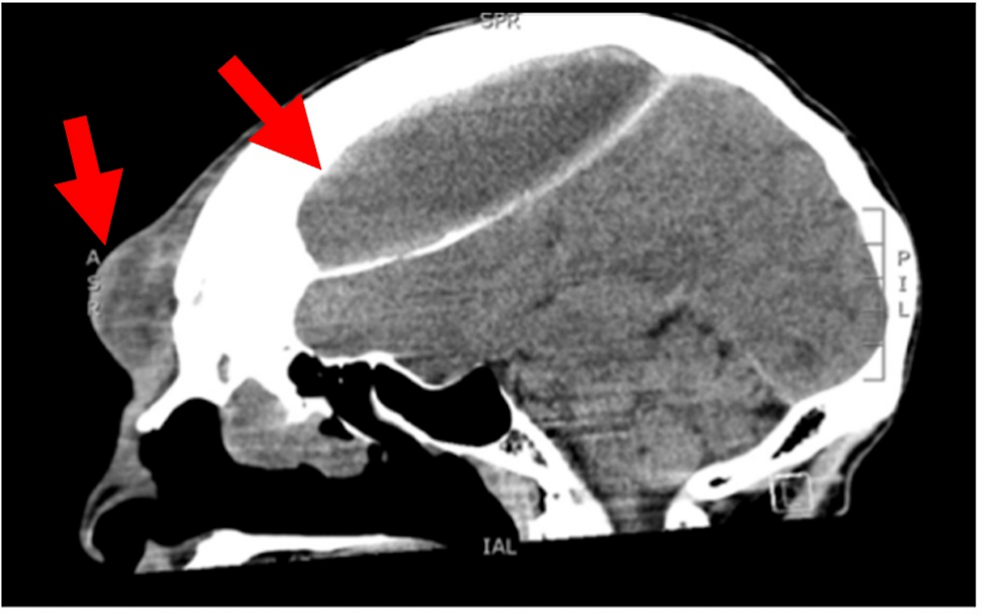
Sagittal View of CT Head and Facial Bones

**Figure 2:**
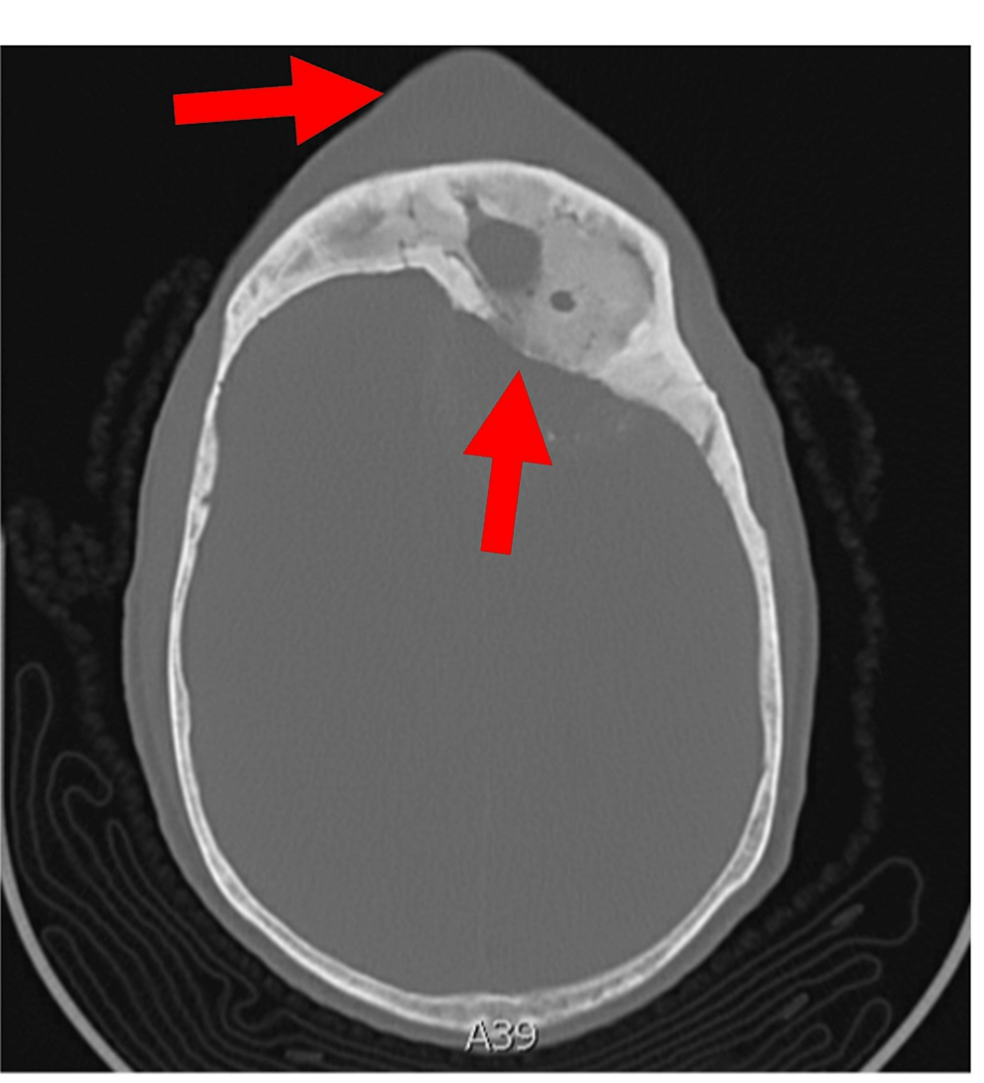
Transverse View of CT Head

**Figure 3:**
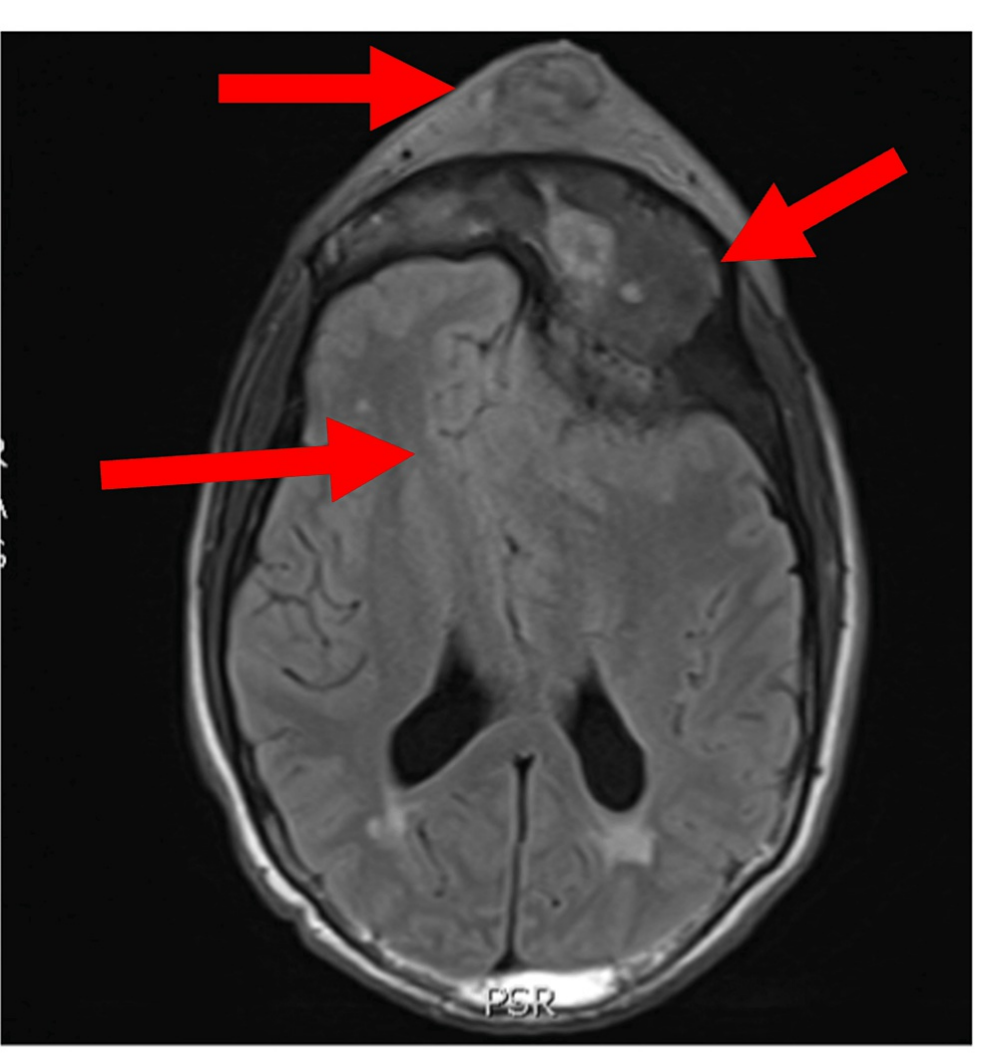
Transverse View of MRI Brain

The patient's vitals were stable; he was afebrile, did not require supplemental oxygen, was normotensive, and had a normal heart rate and rhythm. Laboratory studies revealed a leukocytosis to 13.7x10^3/uL (normal reference range 3.8-10.6x10^3/uL). Physical examination revealed a fluctuant forehead mass, pronounced periorbital edema, copious nasal drainage, and mild ataxia. Though the patient did not meet sepsis criteria, physical examination revealed signs concerning neurologic involvement. CT revealed a frontal mucocele with bony erosion into the subcutaneous forehead and posterior extension into the cranial vault measuring 13 cm anterior-posterior by 5 cm transverse by 4 cm craniocaudal with significant mass effect (Figures 1, 2). Magnetic resonance imaging (MRI) of the head confirmed thick rim-enhancing epidural fluid collection with thickened bone and marked mucus thickening, air-fluid levels in the ethmoid and maxillary sinuses, and partially visualized nasal polyposis. MRI also showed an associated mass effect in the frontal lobes with effacement of the suprasellar cistern and a 1.2 cm rightward midline shift anteriorly (Figure 3). Neurosurgery was emergently consulted and empiric ampicillin-sulbactam was started at a dose of 3 g intravenous infusion every six hours. Aspiration and culture of the subcutaneous portion grew group C streptococci and viridans group streptococci. Given the level of mass effect, cerebral angiography was performed to evaluate for vascular compromise. Cerebral angiography showed no compromise to the well-developed blood supply at the frontal sinus via the large ethmoidal arteries from the left and right ophthalmic arteries, and no arteriovenous fistula was seen. 

Initial neurosurgical consultation recommended transfer to a facility with a higher level of care as the primary facility did not have in-house otolaryngology services. The preferred plan would have been to perform cranial washout by neurosurgery along with simultaneous endoscopic sinus debridement by otolaryngology in the operating room. Despite multiple attempts to transfer, the patient ultimately was not able to seek a higher level of care and underwent neurosurgery alone. The patient underwent a bi-frontal craniotomy with drainage of the abscess and construction of a pericranial flap to create a physical barrier between the obliterated frontal sinus and the cranium. The patient tolerated the procedure well and was ultimately discharged to a post-acute care facility to continue recovery and complete six weeks of intravenous ceftriaxone and metronidazole for intraoperative cultures which grew group C streptococci and *Prevotella denticola*. Neurosurgery recommended that the patient have a repeat CT scan and clinic follow-up approximately one month following the completion of antibiotic therapy. Unfortunately, the patient was lost to follow-up once again.

## Discussion

Mucoceles are slow-growing lesions that typically develop in the paranasal sinuses due to chronic obstruction of the ostia that connects the sinus to the nasal cavity [7]. The frontal sinuses are most frequently involved (60 to 65%), then ethmoid (20 to 30%) and maxillary (10%), with sphenoid sinuses (2 to 3%) having the least involvement [1]. Some inciting factors include mucosal hyperplasia, chronic allergy, inflammation, fibrosis, scarring, neoplasms, and most commonly, chronic rhinosinusitis [2,3,8]. Continued production and accumulation of mucus results in gradual erosion and remodeling of osseous tissue [1]. If left unchecked, pressure-induced necrosis can lead to the destruction of adjacent bony structures. Because the posterior wall of the frontal sinus is notably thin and susceptible to erosion, this can lead to intracranial extension [3,9].

Intracranial extension is a rare complication of chronic rhinosinusitis and mucoceles [10]. Symptoms are commonly subtle with insidious onset, and patients typically remain asymptomatic until significant orbital edema and frontal sinus pain occur. Frontal lobe pain and headache are the most common presenting symptoms [11-13]. Proptosis, retro-orbital pain, diplopia, and visual disturbances may also be presenting symptoms, which makes a thorough history and physical exam important in determining if intracranial extension should be a concern [6,11]. In some cases, intracranial extension can cause frontal lobe syndrome, in which personality changes can occur as a result of mass effect [6]. Recurrence is noted to be somewhere around 10%, including those who had prior sinus surgery. CT imaging is recommended every two years for at least five years after surgery to monitor for recurrence [6].

Any suspicion of cranial involvement should warrant timely imaging to avoid life-threatening consequences. Imaging studies, particularly CT of the facial bones and head and MRI of the brain help delineate the extent of the mucocele involvement within cranial structures. MRI can help differentiate mucoceles from solid neoplasms more accurately than CT [9]. Distension of the affected sinuses is usually evident with osseous thinning and displacement of cranial structures [13]. In this case, vessel imaging was used to rule out any vascular compromise in surgical planning. MRA imaging can be useful in the setting of vascular compromise, particularly in patients with known carotid disease, a history of cerebrovascular disease, or chronic uncontrolled hypertension [9].

A crucial component of this patient’s history, which was known at the time of presentation, was that the patient had regular intranasal cocaine use. The incidence of cocaine-induced midline destructive lesions is 4.8% in chronic cocaine abusers [14]. Chronic intranasal administration of cocaine causes irritation and atrophy of the nasal mucosa as well as necrosis of the cartilage. Common presentations of this damage include epistaxis, nasal septal perforation, saddle nose or alar deformities, hard palate destruction, and sinus erosion [15-17]. Cocaine also slows nasal mucociliary transport, which further contributes to the development of chronic and progressive mucoceles [17]. A known mucocele in the setting of continued structural damage from intranasal cocaine use creates the perfect storm for superinfection or abscess, as with this patient who developed an intracranial extension with epidural abscess. When counseling these patients, it is important to emphasize the education of cocaine cessation and caution in caring for the oropharyngeal mucosal tissue. Education of the risk of repeated cocaine use and identification of social determinants of health both contribute to the ability of a patient to maintain clinical contact and continue surveillance for recurrence.

Traditionally, evacuation and complete excision of the mucocele with neurosurgery was the treatment of choice [13]. As technology has advanced, endoscopic sinus surgery has emerged as a more effective, less invasive method [9]. Successful outcomes have been seen with low rates of recurrence; noted to be down to 3.5% in reviewing endoscope sinus surgery [2]. Current literature states the choice of surgical approach is still contingent on the degree of cranial fossa infiltration and drainage accessibility of the intracranial component [9]. Depending on the degree of intracranial involvement, osteoplastic flaps may be needed to reconstruct the skull base defect [12]. For this patient with frontal sinus mucocele complicated by epidural abscess and neurologic symptoms of ataxia, there was a significant level of intracranial extension and mass effect. Along with his history of chronic rhinosinusitis, cocaine use, and leukocytosis, current literature is consistent with an open surgical approach to adequately eradicate the lesion and drain the intracranial portion of the mucocele.

An important consideration in the clinical course of diagnosing and treating mucoceles is the availability of ophthalmology, otolaryngology, and neurosurgical services at the institution. In this case, the primary facility did not have in-house ophthalmology or otolaryngology services. This is the reality for many hospital centers in the country especially in rural areas. In this patient, two neurosurgeons created a pericranial flap during a bi-frontal craniotomy. They were able to drain the abscess and a portion of the mucocele, using the flap as a physical barrier to help prevent recurrence. This patient would have benefited most from simultaneous otolaryngologic and neurosurgical intervention for combined craniotomy and complete sinus washout to adequately drain, excise, and aim for complete eradication of the infection [9]. A combined approach would also likely have reduced the rate of recurrence with the inclusion of sinus washout. In facilities where all services are available, combined surgical planning can reduce the need for multiple surgeries including reducing the risk of anesthesia. The ability to collaborate inside one institution can also help with record keeping, reducing unnecessary testing and imaging with radiation exposure, as well as optimizing the opportunity to make contact in follow-up. Unfortunately, this patient was again lost to follow-up, so there is no report of any incidence of recurrence in this specific case.

In areas where neurosurgery or otolaryngology are not available, it is vital to recognize the signs and symptoms that would warrant emergent imaging or intervention and subsequent transfer to a higher level of care. As mentioned above, some of these signs would include proptosis, diplopia, subcutaneous forehead mass, fever, purulent nasal drainage, or focal neurologic deficit [6,11-13]. Medical management must also not be delayed. There is no current role for intravenous steroid use presented in the current literature [1]. Empiric use of antibiotics should include agents with empiric coverage for oropharyngeal opportunistic pathogens; ampicillin-sulbactam is often the antibiotic of choice. Cultures should be obtained when able, with infectious disease consultation considered in the case of rare or multi-drug resistant pathogens. Lastly, in cases with eye involvement, ophthalmologic consultation is also warranted [6].

## Conclusions

In patients with known sinus pathology who present with symptoms of forehead swelling and rhinorrhea, especially in the setting of chronic cocaine use, high importance should be given to cranial imaging. Intranasal cocaine use alone puts patients at high risk of sinus pathology from bony and cartilaginous erosion. Any pre-existing damage from cocaine use along with signs of infection could indicate intracranial extension, superinfection, or abscess, for which emergent imaging and subsequent timely consultation of specialists is indicated. In non-emergent cases, endoscopic surgery and continued follow-up imaging will result in the prevention of intracranial extension and reduce rates of recurrence. Medical management with antibiotics and continued monitoring is important to screen for the development of further complications. It is crucial to consider the practice environment, clinical condition, signs of systemic infection, and surgical assessment to determine how best to proceed. In the setting of intracranial extension with neurologic symptoms, such as ataxia or gait instability, as in this patient, a combined neurosurgical and otolaryngologic approach should be implemented to navigate complex infection and intervene for anatomical clearance of infection. 
